# Peripheral immune profile of children with *Talaromyces marneffei* infections: a retrospective analysis of 21 cases

**DOI:** 10.1186/s12879-021-05978-z

**Published:** 2021-03-20

**Authors:** Qiang Zeng, Yingkang Jin, Genquan Yin, Diyuan Yang, Wenyan Li, Tingting Shi, Gen Lu, Li Huang, Huifeng Fan

**Affiliations:** 1 Department of Respiratory Infection, Guangzhou Medical University, No.9, Jinsui Road, Zhujiang New City, Tianhe District, Guangzhou, 510120 Guangdong China; 2 Pediatric Intensive Care Unit, Guangzhou Women and Children’s Medical Center, Guangzhou Medical University, No.9, Jinsui Road, Zhujiang New City, Tianhe District, Guangzhou, 510120 Guangdong China

**Keywords:** *Talaromyces marneffei*, Complication, Immunity, Primary immunodeficiency diseases, Children

## Abstract

**Background:**

*Talaromyces marneffei* (*T. marneffei*) is an opportunistic pathogen that infects immunodeficient children. The aim of the study is to determine the clinical features and peripheral immune state of *Talaromyces marneffei (T. marneffei)* infections in children for early detection and diagnosis.

**Methods:**

We retrospectively reviewed 21 pediatric patients who were diagnosed with *T. marneffei* infections and were followed up in the Guangzhou Women and Children’s Medical Center from January 2010 to January 2020. For each patient, we collected and analyzed clinical characteristics, peripheral immunological results, genetic tests, complications and prognosis.

**Results:**

Common clinical features of the patients included fever (20/21, 95.24%), cough (17/21, 80.95%) and hepatomegaly (17/21, 80.95%). Severe complications included septic shock (12/21, 57.14%), hemophagocytic lymphohistiocytosis (HLH) (11/21, 52.38%), acute respiratory distress syndrome (ARDS) (10/21, 47.62%), multiple organ dysfunction syndrome (MODS) (9/21, 42.86%), and disseminated intravascular coagulation (DIC) (7/21, 33.33%). Eleven children (11/21, 52.38%) eventually died of *T. marneffei* infections. All patients were HIV negative. Seven cases revealed reduced antibody levels, especially IgG. Higher levels of IgE were detected in 9 cases with an obvious increase in two patients. Ten patients showed decreased complement C3 levels, some of whom had low C4 levels. Three patients displayed decreased absolute T lymphocyte counts, including the CD 4+ and CD 8+ subsets. A reduction in NK cells was present in most patients. No patient had positive nitro blue tetrazolium (NBT) test results. Nine patients were screened for common genetic mutations. Of the cases, one case had no disease-specific gene mutation. Four children had confirmed hyperimmunoglobulin M syndrome (HIGM) with *CD40LG* variation, one case had severe combined immunodeficiency (SCID), and one case had hyper-IgE syndrome (HIES). One patient was identified as having a heterozygous mutation in *STAT3* gene; however, he showed no typical clinical manifestations of HIES at his age. One patient had a mutated *COPA* gene with uncertain pathogenic potential. Another patient was diagnosed with HIES that depended on her clinical features and the National Institutes of Health (NIH) scoring system.

**Conclusions:**

*T. marneffei* infections in HIV-negative children induced severe systemic complications and poor prognosis. Children with *T. marneffei* infections commonly exhibited abnormal immunoglobulin levels in peripheral blood, particularly decreased IgG or increased IgE levels, further suggesting possible underlying PIDs in these patients.

**Supplementary Information:**

The online version contains supplementary material available at 10.1186/s12879-021-05978-z.

## Background

*Talaromyces (*formerly *Penicillium) marneffei (T. marneffei)* is an opportunistic pathogen that infects immunodeficient patients in Southeast Asia as a dimorphic fungus. The fungus was first isolated from the hepatic lesions of a bamboo rat (*Rhizomys sinensis*) that died spontaneously from the infection in 1956 [1]. Subsequent studies showed that bamboo rats (*Rhizomys sp*. and *Cannomys sp*.) and soil from their burrows were important enzootic and environmental reservoirs of *T. marneffei*, respectively [2]. Generally, *T. marneffei* presented either as a local or disseminated infection. The symptoms and signs of disseminated *T. marneffei* typically include fever, weight loss, fatigue, hepatosplenomegaly, lymphadenopathy, respiratory, and gastrointestinal abnormalities, and is associated with serious complications and high death rates [3]. In adults, *T. marneffei* infection has been considered to be exclusively associated with acquired immunodeficiency syndrome (AIDS) caused by human immunodeficiency virus (HIV) infection [4]. Therefore, the close correlation between disease manifestation and severity with CD4+ cell count confirms the importance of cell-mediated immunity against *T. marneffei* in adults [2, 5].

The proportion of patients aged 3 months to 16 years among reported cases of *talaromycosis*, is approximately 6–7.5% [6–8]. Unlike the previous reports with most HIV-infected pediatric cases, recent studies showed most pediatric patients were HIV negative [6, 8].. At present, the research on the peripheral immune profile is less comparatively in HIV-negative children with *T. marneffei* infections. In the previous study, the HIV-negative children had a reduction in the number of T-lymphocytes or cellular immunity is probably the most important predisposing factor for *T. marneffei* infection [6]. However, the current knowledge gaps focus on the immune statuses of these children and the categories of primary immunodeficiencies (PIDs) associated with *T. marneffei* infections.

This retrospective study of 21 HIV-negative children who were infected by *T. marneffei* preliminarily assess the immune profile of these patients and to provide insights into the immunological characteristics of the disease in children.

## Methods

A retrospective cohort study was conducted from January 2010 to January 2020 at Guangzhou Women and Children’s Medical Center. Twenty-one children enrolled in this study who presented with culture and/or histopathologically proven infections caused by *T. marneffei*. All data were collected using a standardized form that was based entirely on the medical reports of each patient. The data included demographic information, domiciles, medical history, clinical manifestations, immunologic detection, genetic tests, complications and prognosis.

The diagnostics of PIDs were performed based on clinical characteristics and genetic tests according to the updated classification of PIDs by the Primary Immunodeficiency Expert Committee (PID EC) of the International Union of Immunological Societies (IUIS) [9]. As noted, the National Institutes of Health (NIH) developed a clinical Hyperimmunoglobulin E Syndromes (HIES) scoring system [10], which can serve as a valuable reference for the diagnosis of HIES. The diagnostic criteria included increased IgE (> 1000 IU/mL) plus a weighted score of clinical features > 30 as diagnosis of autosomal dominant HIES (AD-HIES) [11].

This study protocol was conducted in accordance with the Declaration of Helsinki and approved by the Ethics Committee of Guangzhou Women and Children’s Medical Center of Guangzhou Medical University. We obtained all written informed consent from the children’s parents or legal guardians for the use of their clinical and laboratory data from their medical reports.

## Results

### Clinical characteristics

The clinical features of the children are summarized in Table 1 and S1. There were 15 boys and 6 girls with ages ranging from 3 months to 156 months old (median age of 22 months). All patients lived in southern China, though the diagnoses were made throughout the year. The median time from the onset of symptoms to hospitalization was 20 days in our hospital (IQR:12–30 days). The most common clinical presentations of *T. marneffei* infections were fever (20/21, 95.24%), cough (17/21, 80.95%) and hepatomegaly (17/21, 80.95%) (Table 1). Life-threatening complications during hospitalization included septic shock (12/21, 57.14%), hemophagocytic lymphohistiocytosis (HLH) (11/21, 52.38%), acute respiratory distress syndrome (ARDS) (10/21, 47.62%), multiple organ dysfunction syndrome (MODS) (9/21, 42.86%) and disseminated intravascular coagulation (DIC) (7/21, 33.33%). Most patients (19/21, 90.48%) were confirmed by blood culture, and 8 of them had also been confirmed by bone marrow culture. In addition, four cases underwent lymph node biopsy, two cases underwent airway mucosal biopsy and one case underwent skin biopsy. Two special cases confirmed by sputum culture and bronchoalveolar lavage fluid (BALF). Eleven children (11/21, 52.38%) eventually died from *T. marneffei* infections.

**Table 1:** Clinical characteristics of the children with *T. marneffei*

Clinical characteristics	Cases (*N* = 21)
**Demographics**	
Age (months), median (IQR)	22 (15–34)
Gender, male (%)	15 (71.43)
Endemic area, No(%)	21 (100.00)
Month of diagnosis, median (IQR)	7 (4–9)
**Signs and Symptoms**	
The interval from onset to addmission (days), median (IQR)^a^	20 (12–30)
Fever, No. (%)	20 (95.24)
Cough, No. (%)	17 (80.95)
Hepatomegaly, No. (%)	17 (80.95)
Lymphadenopathy, No. (%)	15 (71.43)
Splenomegaly, No. (%)	12 (57.14)
Skin lesion, No. (%)	9 (42.86)
Dyspnea, No. (%)	9 (42.86)
Diarrhea, No. (%)	6 (28.57)
Weight loss, No. (%)	6 (28.57)
Malnutrition, No. (%)	4 (19.09)
Ascites, No. (%)	1 (4.76)
Trachyphonia, No. (%)	1 (4.76)
**Significant complication**	
Sepsis shock, No. (%)	12 (57.14)
HLH, No. (%)	11 (52.38)
ARDS, No. (%)	10 (47.62)
MODS, No. (%)	9 (42.86)
DIC, No. (%)	7 (33.33)
**Specimens for diagnosis**	
Blood, No(%)	19 (90.48)
BM, No(%)	8 (38.10)
Lymph nodes, No(%)	4 (19.09)
Airway mucosal biopsy, No(%)	2 (9.52)
BALF, No(%)	2 (9.52)
Ascites, No(%)	1 (4.76)
Skin, No(%)	1 (4.76)
Stool, No(%)	1 (4.76)
**Antifungal therapy**	
The interval from onset to antifungal therapy (days), median (IQR)	25 (17.5–31.5)
Total, No(%)	19 (90.48)
Amphotericin B, No(%)	8 (38.10)
Voriconazole, No(%)	8 (38.10)
Itraconazole, No(%)	6 (28.57)
Fluconazole, No(%)	5 (23.81)
Caspofungin, No(%)	3 (14.29)
**Outcomes**	
Length of stay (days), median (IQR)	14.5 (4–31)
Mortality, No. (%)	11 (52.38)

*Abbreviation*: *ARDS* acute respiratory distress syndrome, *MODS* multiple organ dysfunction syndrome, *HLH* hemophagocytic lymphohistiocytosis, *DIC* disseminated intravascular coagulation, *BM* bone marrow, *BALF* bronchial alveolar lavage fluid, *IQR*: inter-quartile range

^a^The course of disease before addmission at our hospital

### Immunologic detection

The immunologic detection and genetic tests at the time of diagnosis are shown in Table 2 and S2. All patients were HIV negative according to a serum-specific antibody test. The lymphocyte count, the content of immunoglobulin and complement, and nitro blue tetrazolium (NBT) test in peripheral blood were detected in all cases except one. Among them, seven patients had reduced immunoglobulin content (mainly IgG) (P2, 4, 10, 11, 12, 13 and 17). It is with great regret that 9 children were treated with intravenous immunoglobulin (IVIg) administration before the examination. There were another 9 patients who showed higher levels of IgE (P1, 5, 6, 7, 14, 15, 19, 20 and 21). In particular, IgE levels were obviously increased in two cases (P7 and 21). Low complement C3 levels were found in 10 cases (P1, 2, 5, 6, 7, 8, 9, 12, 18 and 20), and five cases exhibited simultaneously low complement C4 levels (P1, 5, 6, 9 and 20). Of these cases, 9 were fatal. Only three cases presented decreasing T lymphocyte counts, including CD 4+ and CD 8+ subsets, in the results (P8, 12 and 18). P3 revealed only lower CD 8+ subsets. Greater than half of all cases (P1, 5, 7, 8, 11, 12, 13, 14, 15, 18, 19, 20 and 21) showed markedly decreased NK cell counts. The ratio of CD4/CD8 increased in 8 patients (P1, 3, 4, 6, 7, 9, 10 and 17). No one was positive in NBT test.

**Table 2:** Immunoassays findings of the children with *T. marneffei*

Immunoassays findings	Cases (*N* = 21)
**Immunoglobulin**^a^	
Pan-hypogammaglobulinemia (IgG, IgA and IgM), No(%)	1 (4.76)
IgG decrease, No(%)	7 (33.33)
IgA decrease, No(%)	5 (23.81)
IgM decrease, No(%)	1 (4.76)
IgM increase, No(%)	6 (28.57)
IgE increase, No(%)	9 (42.86)
**Complements**	
C3 decrease, No(%)	10 (47.62)
C4 decrease, No(%)	5 (23.81)
**Lymphocyte counts**	
T lymphocyte counts decrease (CD4+ and CD8+), No(%)	3 (14.28)
CD4+ subsets decrease, No(%)	3 (14.28)
CD8+ subsets decrease, No(%)	4 (19.09)
The ratio of CD4/CD8 increase, No(%)	8 (38.10)
NK cells decrease, No(%)	13 (61.90)
**NBT tests**	
Normal, No(%)	21 (100.00)
**HIV detection**	
Negative, No(%)	21 (100.00)

*Abbreviation*: *Ig* immunoglobulin, *NK cells* natural killer cells, *NBT test* nitro blue tetrazolium test, *HIV* human Immunodeficiency virus

^a^Nine children were treated with intravenous immunoglobulin (IVIg) administration before the examination

### Genetic tests and primary deficiencies

For personal and economic reasons, nine patients underwent gene sequencing, three patients(P3, 9 and 19) by whole exome sequencing, six patients(P4,10, 16,17,18 and 21) by selective PID panel testing, seven of whom were identified with specific gene variation, one patient (P9) with uncertain gene variation, and one patient (P3) without disease-specific gene mutation (Table 3). Four children (P4, 10, 16 and 17) were confirmed to have hyperimmunoglobulin M syndrome (HIGM) because they identified mutations or microdeletions in the *CD40LG* gene. A patient (P18) was diagnosed with severe combined immunodeficiency (SCID) due to an *IL2RG* mutation. Another patient (P21) was diagnosed with hyper-IgE syndrome (HIES) based on his NIH score and *STAT3* mutation, and the NIH score was 48 (Table S3). P19 had yet to exhibit typical clinical manifestations of HIES at his age. His NIH score was 20 although he had a heterozygous mutation in *STAT3* gene. The last gene (P9) has a mutated *COPA* gene with uncertain pathogenic potential. Because the other 12 patients did not undergo genetic tests, their PIDs were not determined. In particular, P7 was diagnosed with HIES that depended on her clinical features and the NIH scoring system, and her score was 56.

**Table 3:** Genetic detection and primary immunodeficiencies of the children with *T. marneffei*

Patient	Gene	Genetic variation	Nucleotide variation	PIDs	Gene testing
3	Not Found	Not Found	Not Found	Not Determined	Whole exome gene sequencing
4	*CD40LG*	Hemizygote	c.424_436del	HIGM	PID genes panel
7	Not done	–	–	HIES^a^	–
9	*COPA*	Heterozygous	c.2437G > T	Not Determined	Whole exome gene sequencing
10	*CD40LG*	Microdeletion	> 132 kb	HIGM	PID genes panel
16	*CD40LG*	Microdeletion	c.1978 + 1G > A	HIGM	PID genes panel
17	*CD40LG*	Hemizygote	c.598A > T	HIGM	PID genes panel
18	*IL2RG*	Hemizygote	c.185G > A	SCID	PID genes panel
19	*STAT3*	Heterozygous	c.1679_1681del	Not Determined^b^	Whole exome gene sequencing
21	*STAT3*	Heterozygous	c.A1593T	HIES	PID genes panel

*Abrreviation*: *PIDs* primary immunodeficiencies, *HIGM* hyperimmunoglobulin M syndrome, *HIES* hyper-IgE syndrome, *SCID* severe combined immunodeficiency, *NIH* National Institutes of Health

^a^The patient was diagnosed with HIES based on her clinical features and the NIH scoring system

^b^The NIH score of this patient remained substandard to confirmed HIES at his age

## Discussion

Talaromyces (formerly *Penicillium*) marneffei*(T. marneffei)* is an opportunistic pathogen that infects immunodeficient children. The aim of the study is to determine the clinical features and peripheral immune state of *T. marneffei* infections in children for early detection and diagnosis. *T. marneffei* is an emerging pathogenic fungus that can cause fatal systemic mycosis in immunocompromised hosts and occurs mostly in humid tropical climate regions, including the south and southwest regions of China [12]. In adult, *T. marneffei* infection occurs mainly in AIDS patients, where it is a severe, deep mycosis with high mortality [4]. In contrast to adults, subjects with PIDs are more susceptible in children according to previous reports [6, 8]. Furthermore, the recognition of immune status by clinical observation is important to treat and prevent *T. marneffei* infections in children and to facilitate the diagnosis and reporting of PIDs. We retrospectively analyzed the immunity status of 21 children with *T. marneffei* infection over this decade, including the immunoglobulin pattern (IgG, IgA, IgM, and IgE) and enumeration of lymphocyte subpopulations (T-, B-, and NK-cells) in peripheral blood. We found that abnormal immunoglobulin findings were considerable in HIV-negative children with *T. marneffei* infection, mainly in those with decreased IgG or increased IgE.

In our study, we summarized 21 documented pediatric cases of proven *T. marneffei* infection in southern China. Most cases presented with fever, weight loss, swollen lymph nodes, generalized lymphadenopathy, and hepatomegaly. In many aspects, the clinical manifestations of pediatric patients are different from those of adult patients [6, 8, 13]. For example, respiratory system involvement was also observed in most cases, but skin lesions were unusual in pediatric patients in the present study. A high frequency (70–80%) of skin lesions has been reported in adults with *T. marneffei* infection [14]. Nonspecific clinical manifestations are a potential cause of misdiagnosis for *T. marneffei* infection. *T. marneffei* infection is a severe disease that can lead to high mortality rates of greater than 50% in children in previous reports [6]. Of these cases, 11 were fatal, and all fatal cases in our study died of serious complications, such as HLH, septic shock, MODS, DIC and ARDS. It is noteworthy that the incidence of HLH in pediatric patients was significantly higher than that in adults, possibly because many of our children had delayed treatment due to early misdiagnosis. Another major cause of complications and high mortality in HIV-negative patients with *T. marneffei* infection may be abnormal host immune function.

In the present study, all of these children were HIV negative, but they almost had abnormal immune parameters at the time of diagnosis, especially in the immunoglobulin contents, including decreased levels of IgG or increased levels of IgE in peripheral blood. However, in only three cases, the number of peripheral blood lymphocytes significantly decreased. Previous studies have suggested that a reduction in the number of T lymphocytes or cellular immunity is probably the most important predisposing factor for adult *T. marneffei* infection [6, 7]. Serum antibody levels were increased in HIV-negative adults in one study [15]. Unlike adults, the decreased levels of IgG in peripheral blood were found in 7 cases. There were 9 cases with increased IgE, two of them had markedly elevated IgE levels. Taking into account that 9 cases were treated by IVIg prior to the immune detection being performed, which certainly influenced immunoglobulin levels in peripheral blood. Even so, the common immunoglobulin changes in children with *T. marneffei* infection are lower IgG or higher IgE in peripheral blood. That is, pediatricians should be alert to *T. marneffei* infection in children when peripheral immunoglobulin changes.

On the other hand, abnormal immunity may occur secondary to serious *T. marneffei* infection. Previous studies focused less on complement levels. In our study, approximately half of all patients displayed decreased complement levels, especially C3. It should be noted that of the 11 deaths of our children, 9 cases of complement dramatically declined. The complement system has a determinant role in defense against infections [16], so the reduction in complement is probably also an important index of children with severe *T. marneffei* infection. In addition, reduced NK cell counts were identified in most patients. NK cells are the prototype innate lymphoid cells endowed with potent cytolytic functions that provide host defense against microbial infection [17]. The reduction in NK cells may be due to HLH secondary to severe *T. marneffei* infection. Further studies are necessary to evaluate immunologic parameters to explore the influence of body immunity from severe *T. marneffei* infection.

Abnormal immunological findings might suggest underlying PIDs in HIV-negative children with *T. marneffei* infection. This point has been reported in children with various forms of immune-related underlying diseases and PIDs [7, 18, 19]. As a further study of *T. marneffei* infection, we performed nine genetic tests of underlying PIDs in recent years. The results showed that four cases were identified as HIGM with mutations or microdeletion of the *CD40LG* gene. There were two patients with mutations in *STAT3* gene, one of whom was diagnosed with HIES in combination with typical clinical features. We have not diagnosed HIES in another child now, considering many clinical signs did not appear at his age (1 year old). His NIH score was only 20, but it was not completely excluded. A hemizygous mutation of *IL2RG* was identified in one patient diagnosed with SCID. The immunoassay results of the patient displayed reduced numbers of lymphocyte subsets along with remarkable declines in immunoglobulin levels. This finding is consistent with *IL2RG* mutation for definitive diagnosis. One patient had a mutated *COPA* gene with uncertain pathogenic potential. Patients with *COPA* mutations typically have normal numbers and percentages of lymphocytes and lymphocyte subsets along with unremarkable immunoglobulin levels and intact production of specific antibodies [20]. However, the child had remarkedly decreased immunoglobulin with normal numbers of lymphocytes. The exact mechanism by which *COPA* gene mutation causes *T. marneffei* infection is currently unknown. Although no genetic testing was performed on 12 patients, some of them showed degrees of abnormal immunity in peripheral blood. Of note is patient 7. She was diagnosed with HIES based on her clinical features and the NIH scoring system, but she did not receive genetic tests. In addition, P2, P11, P12 and P13 were accompanied by a decrease in IgG levels, and P12 coincided with obviously reduced counts of lymphocyte subsets. This finding might suggest that these patients could have had potentially underlying immunodeficiencies that were not identified or that their poor prognosis was linked to a more systemic impairment in immunity.

The study still has some limitations that should be considered. This is a retrospective single-center analysis, and some earlier HIV-negative children with *T. marneffei* did not receive genetic tests; hence, the proportion of such cases with underlying PIDs is unknown. In addition, serum neutralizing anti-IFN-γ autoantibodies has not yet been carried out in our hospital, it is with great regret that our patients failed to get tested. Furthermore, anti-IFN-γ autoantibodies associated immunodeficiency is partly due to inherited mutations in IFN-γ-signaling-associated factors, however we such mutations were not detected in our small cohort. We will pay more attention to the disease and perform the testing in the future. Nevertheless, this study may provide a valuable reference for immunity monitoring in children with *T. marneffei* infection.

## Conclusions

*T. marneffei* infections in HIV-negative children involve many systemic complications, high mortality and poor prognosis. There were obvious immunoglobulin abnormalities in these children with severe *T. marneffei* infections in peripheral blood, especially in decreased IgG or increased IgE, suggesting possibly underlying PIDs.

## Supplementary Information

**Additional file 1.**

**Additional file 2.**

**Additional file 3.**

## Data Availability

The datasets generated and/or analyzed during the current study are not publicly available due individual privacy of patients could be compromised, but are available from the corresponding author on reasonable request.

## Abbreviations

AIDS: Acquired immunodeficiency syndrome
ARDS: Acute respiratory distress syndrome
BALF: Bronchoalveolar lavage fluid
DIC: Disseminated intravascular coagulation
HIES: Hyper-IgE syndrome
HIGM: Hyperimmunoglobulin M syndrome
HIV: Human immunodeficiency virus
HLH: Hemophagocytic lymphohistiocytosis
IUIS: International Union of Immunological Societies
IVIg: Intravenous immunoglobulin
MODS: Multiple organ dysfunction syndrome
NBT: Nitro blue tetrazolium
NIH: National Institutes of Health
PIDs: Primary immunodeficiencies
PID EC: Primary Immunodeficiency Expert Committee
SCID: Severe combined immunodeficiency
